# RBM15-mediated N6-methyladenosine modification affects COVID-19 severity by regulating the expression of multitarget genes

**DOI:** 10.1038/s41419-021-04012-z

**Published:** 2021-07-23

**Authors:** Yuting Meng, Qiong Zhang, Kaihang Wang, Xujun Zhang, Rongwei Yang, Kefan Bi, Wenbiao Chen, Hongyan Diao

**Affiliations:** 1 State Key Laboratory for Diagnosis and Treatment of Infectious Diseases, National Clinical Research Center for Infectious Diseases, Collaborative Innovation Center for Diagnosis and Treatment of Infectious Diseases, The First Affiliated Hospital, College of Medicine, Zhejiang University, Hangzhou, China; 2 Department of Clinical Engineering and Information Technology, The First Affiliated Hospital, College of Medicine, Zhejiang University, Hangzhou, China

**Keywords:** Infectious diseases, Biomarkers, Infectious diseases

## Abstract

Severe coronavirus disease 2019 (COVID-19) is characterized by symptoms of lymphopenia and multiorgan damage, but the underlying mechanisms remain unclear. To explore the function of N6-methyladenosine (m6A) modifications in COVID-19, we performed microarray analyses to comprehensively characterize the m6A epitranscriptome. The results revealed distinct global m6A profiles in severe and mild COVID-19 patients. Programmed cell death and inflammatory response were the major biological processes modulated by severe acute respiratory syndrome coronavirus 2 (SARS-CoV-2) infection. Further, RBM15, a major m6A methyltransferase, was significantly elevated and positively correlated with disease severity. Silencing RBM15 drastically reduced lymphocyte death in vitro. Knockdown of RBM15 remarkably suppressed the expression levels of multitarget genes related to programmed cell death and inflammatory response. This study shows that SARS-CoV-2 infection alters the m6A epitranscriptome of lymphocytes, particularly in the case of severe patients. RBM15 regulated host immune response to SARS-CoV-2 by elevating m6A modifications of multitarget genes. These findings indicate that RBM15 can serve as a target for the treatment of COVID-19.

## Introduction

The outbreak of coronavirus disease 2019 (COVID-19) caused by severe acute respiratory syndrome coronavirus 2 (SARS-CoV-2) has led to major concerns worldwide [1–3]. The clinical manifestations of COVID-19 are extremely heterogeneous, varying from asymptomatic illness to significant hypoxia with severe acute respiratory distress syndrome and multiple organ failure [4–6]. The mechanism that accounts for lymphopenia and multiorgan damage remains unclear, and a mere single-target therapy cannot be expected to achieve desirable curative effects. Exploring underlying pathogenesis and developing treatment measures have become an urgent need across the world.

COVID-19 involves not only the activation of antiviral immune responses but also uncontrolled inflammatory responses, leading to an impaired immune system and aberrant production of cytokines, lymphopenia, and lymphocyte dysfunction [7–10], which could be attributable to SARS-CoV-2-induced apoptosis of lymphocytes [11–14]. The molecular mechanisms underlying the development of lymphopenia in such cases remain poorly understood. In addition, the role of epigenetic remodeling in shaping lymphopenia of SARS-CoV-2 infection remains largely unclear.

N6-methyladenosine (m6A) is widely reported to involve in the immune response and viral infections by altering the expression of innate immune signaling molecules, modulating T-cell homeostasis, and regulating viral replication [15, 16]. The absence of m6A modifications owing to METTL3/METTL14 depletion reportedly upregulates IFN-β expression levels in cases of human cytomegalovirus infection, indicating that m6A modifications modulate the antiviral response by regulating the levels of key molecules involved in the innate immune response [15, 16]. In addition, the m6A modifications of *Socs1* and *Socs3* regulates T-cell differentiation and proliferation by increasing the abundance of suppressors of cytokine signaling family proteins [17]. Therefore, m6A modification seems to be a suitable therapeutic target.

In this study, we performed m6A epitranscriptomic microarray using peripheral blood samples obtained from COVID-19 patients and healthy controls, and determine the altered m6A modification level of lymphocytes. We subsequently screened m6A regulators and performed gene ontology (GO) analysis and gene set enrichment analysis. RNA-binding motif protein 15 (RBM15) overexpression was detected in patients with COVID-19. The principal role of RBM15 is to recruit the methyltransferase complex to the target gene, facilitating the methylation of adenosine nucleotides [18]. Hence, we verified several high-confidence downstream target genes with hypermethylation, including caspase (CASP) 1, CASP5, and tribbles homolog 1 gene (TRIB1), thymic stromal lymphopoietin (TSLP), DEAD-box helicase 3 X-linked (DDX3X), and interleukin 17 receptor B (IL17RB), which facilitate programmed cell death and induce an abnormal inflammatory response in severe patients. In vitro experiments demonstrated that RBM15-mediated regulation of multitarget genes was triggered by treatment with spike (S) protein, but not hemagglutinin (HA) protein. Our results provide a new perspective regarding mechanisms underlying COVID-19 and also provide new insights for developing diagnostic and treatment methods.

## Result

### Global m6A levels are increased in PBMC of COVID-19 patients and showed differentially m6A-modified transcripts

**Fig. 1:**
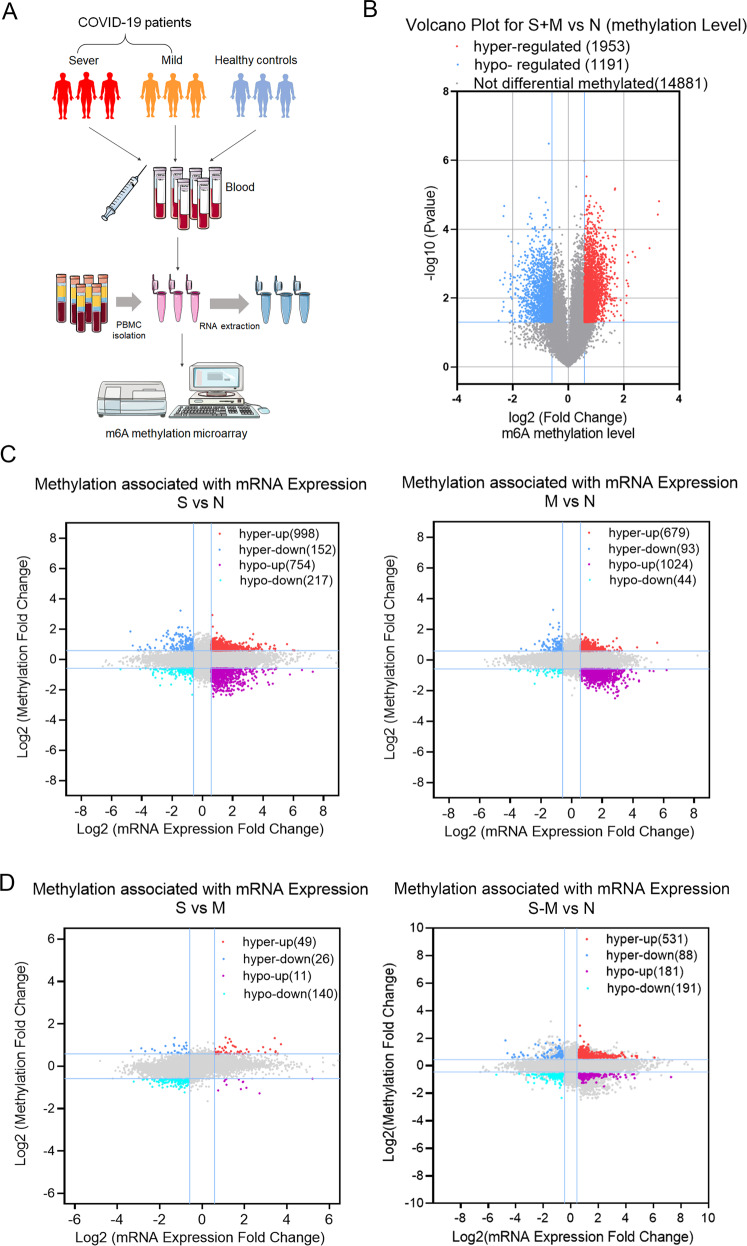
Global m6A levels are increased in PBMC of COVID-19 patients and showed differentially m6A-modified transcripts. **A** Experimental design. **B** Volcano plot showing abnormally methylated mRNAs in group S (left) and M (right), as compared with group N (FC > 1.5, *P* < 0.05). **C** Volcano plots showing the correlation between log2 fold change (FC) of differentially expressed transcripts and log2 FC of differentially m6A-methylated transcripts in groups S and M in comparison with group N separately. **D** Volcano plots of log2 FC of differentially expressed transcripts and aberrantly methylated transcripts in group S in comparison with group M (left) and the figure on the right showed differentially genes only in group S compared with group N (color plots in four quadrants) but not in M compared with N (gray plots in four quadrants) (FC > 1.5, *P* < 0.05).

We herein performed m6A epitranscriptomic microarray using peripheral blood samples obtained from three mild patients (group M), three severe patients (group S), and three healthy controls (group N; Fig. 1A, Supplementary Table 1). Principle component analysis showed distinct clustering of individual samples belonging to the three groups (Fig. S1A, B), indicating the precision and reproducibility of microarray analyses. Our microarray data revealed that in comparison with the m6A levels in group N, 1953 mRNAs were significantly hypermethylated and 1191 mRNAs were hypomethylated in patients with COVID-19, which showed that more transcripts were hypermethylated in COVID-19 (Fig. 1B). Also, the differentially methylated genes (DMGs) of group S and group M were identified separately, the volcano plots and Venn plots indicated there are more hypermethylated genes in severe groups (Fig. S1C). These findings revealed that SARS-CoV-2 infection upregulated global m6A levels, particularly in the case of severe patients. With regard to mRNA expression levels, more genes were upregulated in patients with COVID-19 (Figure S1D). In addition, we assessed the methylation profile and mRNAs expression levels to construct a bivariate map (Fig. 1C). More hypermethylated with upregulated (Hyper-up) genes were identified in Group S (998) and hypomethylated with upregulated (Hypo-up) genes (1024) occupy the main proportion in Group M. To understand the role of m6A methylation in severe patients, we analyzed the genes significantly regulated in group S compared with group M and few genes were identified in this part. This is not consistent with the above differences of DMGs between group S and group M. Then, we compared the transcripts pattern and analyzed the genes regulated in group S (but not in group M). We surprisingly found that more than half (531/998) Hyper-up genes of S were specific (Fig. 1D). These results indicated the highly distinguished methylated and regulated pattern between S and M groups. To summarize, we described the m6A methylation dysregulated pattern in patients with COVID-19 and revealed the relationship between epigenetic and transcriptional signatures in severe and mild patients.

### Aberrant methylation triggered by SARS-CoV-2 regulates multiple targets related to immune response and cell death pathways

**Fig. 2:**
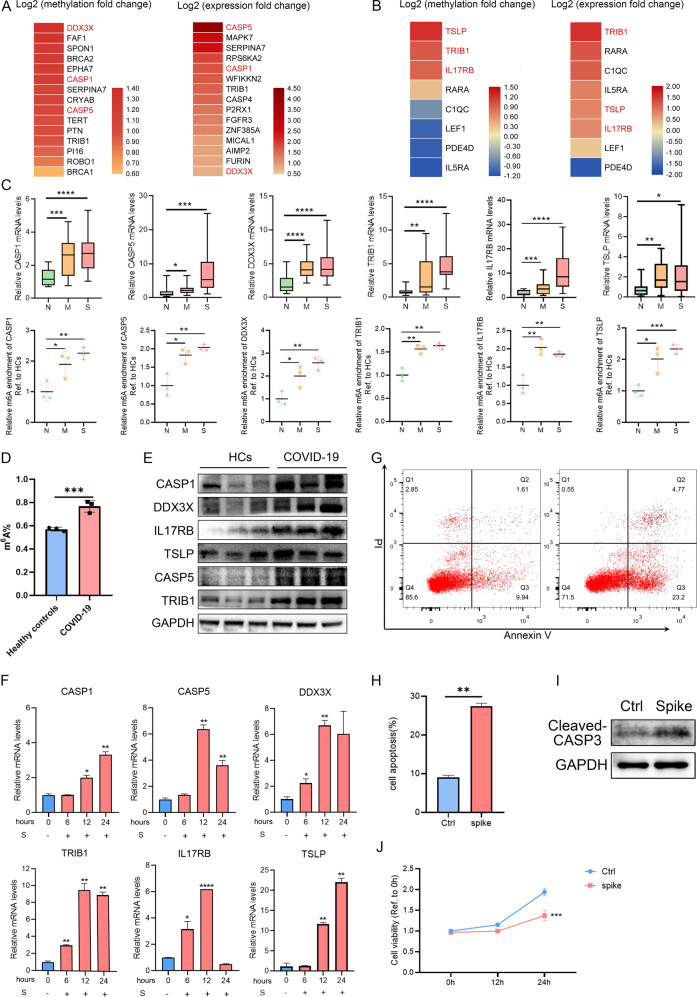
Aberrant methylation triggered by SARS-CoV-2 regulates multiple targets related to immune response and cell death pathways. Heatmap of genes (*P* < 0.01, enrichment factor >1.5) related to the cell death (**A**) and immune response (**B**). Box plot (**C**) depicting the expression levels of CASP1, CASP5, DDX3X, TRIB1, IL17RB, and TLSP in patients with COVID-19 (20 severe and 19 mild patients) as compared with those in healthy controls (*n* = 20). And scatter plots revealed m6A enrichment of CASP1, CASP5, DDX3X, TRIB1, IL17RB, and TSLP in patients. **D** Total m6A levels of COVID-19 patients and healthy controls (*n* = 3). **E** The protein levels of candidate genes were detected by western blot in COVID-19 patients and healthy controls (HCs). **F** CASP1, CASP5, DDX3X, TRIB1, IL17RB, and TLSP expression levels in HuT 78 cells co-cultured with activated THP-1 cells were measured after being treated with S proteins separately for 0, 6, 12, and 24 h. HuT 78 cells were co-cultured with (Ctrl) or without (spike) spike proteins (50 ng/ml), and cell death was detected by apoptosis assay (**G**), quantitative analysis of positive signals was showed in (**H**), and the protein levels of cleaved–CASP3 were detected using western blot (**I**). CCK-8 assay (**J**) was applied to evaluate proliferation abilities of HuT 78 cells with spike stimulated or without spike. Data were shown as means ± SD (*n* = 3, *P < 0.05, **P < 0.01, ***P < 0.001, and ****P < 0.0001).

We identified 711 aberrant methylated genes (fold change (FC) > 2, *P* < 0.05, Supplementary Table 2) in groups S and M in comparison with group N. To further comprehend the functional effects associated with changes in methylation, we used Metascape to perform a pathway and process enrichment analysis. The top 20 GO biological process annotation clusters were shown in Figure S2A and a network plot was rendered in Figure S2B. Among these clusters, the top 2 clusters distinctly abundant in patients with COVID-19 were related to the immune response and cell death. Genes from hierarchical clusters on these two enriched terms were summarized and the changes in m6A methylation and mRNA expression levels were shown in Figs. 2A, B, S2C. Hyper-Up genes were selected (highlighted in red font) and a comparison was performed between groups S and N (*P* < 0.05). We further verified elevating of expression levels, methylation levels (Fig. 2C, S2D), and protein levels (Fig. 2E) of these prominent genes (IL17RB, TSLP, TRIB1, CASP1, CASP5, and DDX3X) (Supplementary Table 3). The increase of total m6A levels in peripheral blood mononuclear cell (PBMC) samples of COVID-19 was also verified (Fig. 2D). In addition, we constructed an in vitro cell model by treating HuT 78 cells (cutaneous T-lymphocyte cell line) co-cultured with activated THP-1 cells with S protein (the main component of SARS-CoV-2) and HA protein from the H1N1 virus. The expression of CASP1, CASP5, TRIB1, TSLP, and DDX3X were specifically upregulated by S protein, but not to HA protein, whereas IL17RB expression level was upregulated by both proteins (Figs. 2F, S2E). Lymphopenia has been reported to be prevalent in severe patients with COVID-19. To explore whether the cell death pathways in the above mentioned contribute to the severity of COVID-19, we performed a cell death assay with statistical analysis (Fig. 2G, H) and detected the protein levels of cleaved–CASP3 (Fig. 2I) after stimulated with a spike. The result showed that spike could induce apoptosis of HuT 78 cells and elevate the expression of cleaved–CASP3. Then, we descript the cell survival curves after spike stimulation using the CCK-8 assay (Fig. 2J). The cell viability of HuT 78 cells with spike stimulation decreased in time-dependent. In sum, these results indicated that m6A methylation exerts a regulatory effect on multiple targets belong to cell death pathways and inflammatory response pathways in patients with COVID-19. In addition, the cell death pathway in lymphocytes could contribute to lymphopenia in severe patients with COVID-19.

### RBM15 is elevated in SARS-CoV-2 infection and positively correlated with the severity of the COVID-19

**Fig. 3:**
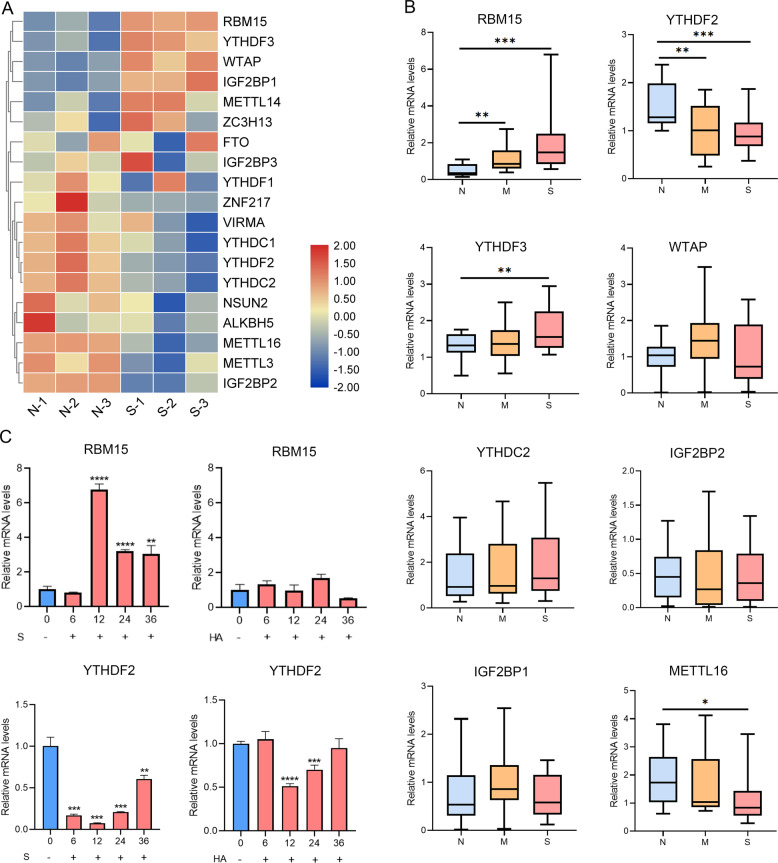
RBM15 is elevated in SARS-CoV-2 infection and positively correlated with the severity of the COVID-19. **A** m6A regulators were selected from microarray data and then analyzed. Heatmap showing the expression of regulators in group S vs N. **B** Box plot showing gene expression results obtained using qRT-PCR (N: 20 healthy controls, M: 19 mild patients, S: 20 severe patients, *P < 0.05, **P < 0.01, and ***P < 0.001). **C** RBM15 and YTHDF2 expression levels in HuT 78 cells co-cultured with activated THP-1 cells were measured after being treated with HA and S proteins separately for 0, 6, 12, 24, and 36 h. Data were shown as means ± SD (*n* = 3, *P < 0.05, **P < 0.01, ***P < 0.001, and ****P < 0.0001).

To elucidate transcript-specific m6A changes induced by SARS-CoV-2 infection, we analyzed m6A regulators in mRNA expression microarray data and evaluated differences of regulators between severe patients and healthy controls (Fig. 3A). We observed that the writers containing WTAP and RBM15 and the readers including YTHDF3 and IGF2BP1 were significantly elevated, while METTL16, YTHDF2, YTHDC2, and IGF2BP2 expression levels were downregulated in severe patients (*P* < 0.01). For further validation of our data, we performed qRT-PCR using peripheral blood samples. We observed the strong upregulation of RBM15 and downregulation of YTHDF2 (Fig. 3B). In addition, the expression of RBM15 was elevated upon S protein treatment, but HA protein treatment had no effect in vitro cell model (Fig. 3C). Considering that more mRNA genes were hypermethylated in patients with COVID-19 (Fig. 1B), we validated that the m6A writer RBM15 was highly expressed in severe COVID-19 patients, having a critical role in modulating the balance of m6A modifications and having important functions in patients with COVID-19.

### RBM15 promoted the expression of functional genes by elevating m6A modification

**Fig. 4:**
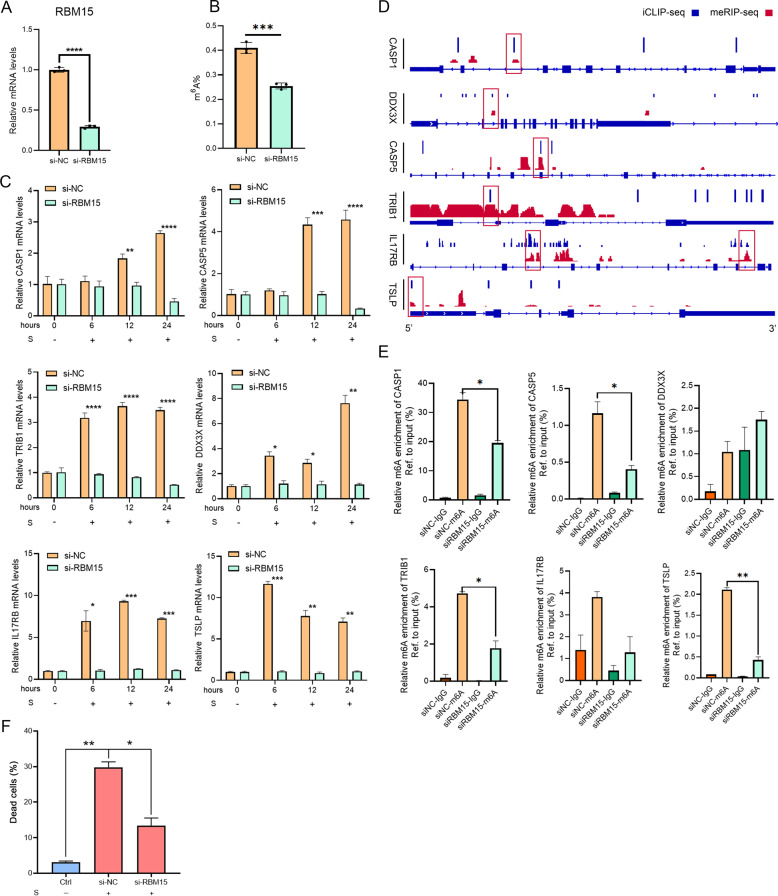
RBM15 promoted the expression of functional genes by elevating m6A modification. HuT 78 cells transfected with either control siRNA (NC) or siRBM15 were cultured. mRNA expression level of RBM15 (**A**) was detected by qRT-PCR and total methylation level (**B**) was detected using a methylation quantification kit. **C** HuT 78 cells co-cultured with activated THP-1 cells transfected with either control siRNA (NC) or siRBM15 were treated with S protein for 0, 6, 12, and 24 h. CASP5, CASP1, and TRIB1 expression levels were detected using qRT-PCR. **D** Analyses of iCLIP-seq data and MeRIP-seq Data were plotted by the IVG. m6A abundance on CASP1, CASP5, DDX3X, IL17RB, TRIB1, and TSLP were aligned to the corresponding reference genome (blue color below figure). Blue colors (In the above) show the m6A signals of iCLIP-seq data, whereas red stands for MeRIP-seq data. At the same position, m6A peaks of iCLIP-seq group overlap MeRIP-seq group were recognized as the genuine m6A location. The most remarkable locations were highlighted with a Red block. **E** m6A modification of CASP1, CASP5, DDX3X, IL17RB, TRIB1, and TSLP were detected by MeRIP-qPCR analysis. Relative m6A enrichment of these genes for each IP group was normalized to input (%). Data were shown as means ± SD (*n* = 3, *P < 0.05, **P < 0.01, ***P < 0.001, and ****P < 0.0001).

To understand the function of RBM15 in SARS-CoV-2 infection and validate the regulatory effects of m6A modification on the aforementioned functional genes, we then knockdown RBM15 in HuT 78 cells (Fig. 4A) induce the decline of total m6A methylation levels (Fig. 4B). We further performed qRT-PCR to assess the mRNA expression level of key genes. HuT 78 cells were transfected with siRBM15 or siNC segments for 8 h and co-cultured with activated THP-1 (with or without spike stimulated), and harvest cells at 0, 6, 12, 24 h. The results showed that the upregulation of CASP1, CASP5, TRIB1, DDX3X, IL17RB, and TSLP stimulated by S protein could be attenuated by silencing RBM15 (Fig. 4C). Considering the principal role of RBM15 is to facilitate the methylation of adenosine nucleotides [18]. We analyzed the individual-nucleotide resolution cross-linking and immunoprecipitation (iCLIP) data set and methylated RNA immunoprecipitation sequencing (MeRIP-seq) data sets and the results showed putative m6A residues in genes above mentioned, some of which are localized at the same region (Fig. 4D). We designed specific primers according to these co-location regions and performed MeRIP-qPCR to verify the regulation of RBM15. Results showed that the m6A levels of CASP1, CASP5, TRIB1, and TSLP were decreased notably after knockdown RBM15, whereas IL17RB has a tendency to downregulate methylation and DDX3X may be regulated by RBM15 through the methylase independence pathway (Fig. 4E). We further performed an apoptosis assay in HuT 78 cells stimulated with S protein. S protein treatment led to cell death of HuT 78 cells, which could be attenuated by silencing RBM15 (Fig. 4F). These results suggest that RBM15 has a key role in promoting functional gene expression through the methylase pathway and inhibits apoptosis of lymphocytes in patients with SARS-CoV-2 infection.

### RBM15 contribute to the severity of COVID-19

**Fig. 5:**
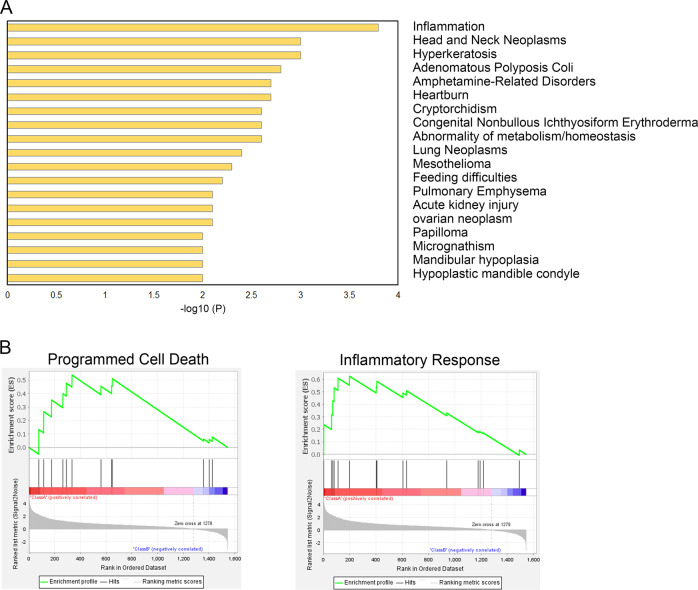
RBM15 contributes to the severity of COVID-19. **A** DisGeNET analysis for Hyper-Up genes specifically present in the severe group. **B** Gene set enrichment analysis (GSEA) was performed using Hyper-Up genes (531) in group S, but not in group M. GSEA was used using the Molecular Signatures Database (MSigDB) of Hallmarks gene sets (h.all.v7.1.symbols), Canonical pathways gene sets (c2.cp.v7.1.symbols) and GO biological process gene sets (c5.bp.v7.1.symbols).

To understand the role of m6A methylation in severe patients, DisGeNET analysis of Hyper-Up genes was performed using Metascape (Fig. 5A), which indicated that inflammation, pulmonary emphysema, and acute kidney injury were related to aberrant m6A methylation, potentially contributing to the aggravation of COVID-19. Gene set enrichment analysis (GSEA) was performed to distinguish potential functions of Hyper-Up genes in severe patients (Fig. 5B). Inflammatory response and programmed cell death were distinctly abundant. It has been widely reported that lymphopenia is commonly observed in severe COVID-19 patients. Our study suggested that the m6A hypermethylation induced gene cluster upregulation, promoting cell death, and accelerating disease progression, which could be alleviated by silencing RBM15.

## Discussion

Despite tremendous medical efforts and extensive analyses of cytokines and cells such as lymphocytes, the mechanism underlying lymphopenia in severe COVID-19 patients remains unclear. In this study, we obtained the epigenetic (m6A) microarray (44122 mRNAs and 12496 long noncoding RNAs) profile using PBMCs obtained from patients with COVID-19. In total, in comparison with group N, 2500 mRNAs in group S and 2068 mRNAs in group M (FC > 1.5, *p* < 0.05) were detected to have significantly different m6A levels. Furthermore, in patients with COVID-19, a higher proportion of mRNAs were significantly hypermethylated which is consistent with the previous reports [19]. Especially, we found the proportion of hypermethylation genes in severe patients was higher than that in mild which showed a potential correlation with disease severity.

We next selected 711 mRNAs with significantly different (FC > 2, *P* < 0.05) m6A methylation levels in patients with COVID-19. GO analysis revealed that the top clusters related to the immune response and cell death were distinctly abundant in patients with COVID-19. Abnormal immune response with T-cell function deficiency was reported to be one of most major features in disease progression in our previous report [20]. In this report, we found that DMGs may affect lymphocyte apoptosis by regulating the cell death pathway. This is another important clue for us to explore the mechanism of the abnormal function of T cells. Moreover, the candidate target genes we identified were also reported to be involved in the cell death pathway. CASP1 has been reported to play an important role in programmed cell death processes [21, 22] and pyroptosis [23]. In addition, according to GO analysis, CASP1 was clustered in the cell death pathway; thus, we speculate that CASP1 has a key role in the programmed cell death of lymphocytes in patients with COVID-19, and this function may be closely related to disease severity. Kamada et al. [24] showed that CASP5 overexpression induced Rat-1 cell apoptosis, whereas Zhu [25] reported that its inhibition reduced hypoxia-induced apoptosis in hepatocytes. Our data strongly indicate that methylase RBM15 leads to the overexpression of CASP1 and CASP5 through the methylase pathway upon treatment with S protein, but not HA protein. These findings suggest that in contrast to other respiratory viral infections such as influenza, RBM15 has an important role in lymphocyte death through regulated functional genes in the case of COVID-19. Thus, inhibition of m6A methylation may alleviate lymphopenia by suppressing the death of lymphocytes.

Further, we found that RBM15 regulates several genes that belong to the immune response pathway in patients with SARS-CoV-2 infection. Among these, TRIB1 reportedly has a fundamental part in the differentiation and proliferation of lymphocytes. A recent study found that TRIB1 overexpression inhibited CD4^+^ T-cell proliferation and restrained the population of KLRG1^+^ effector CD8 T cells [26, 27]. This suggests that there are some functional genes related to the inflammatory response pathway were regulated by m6A methylation in patients with COVID-19. In our study, owing to the limitation of the virus experiment in vitro, we did not elaborate on the mechanism of the effect of m6A on immune response, and a more in-depth mechanism needs to be further explored.

The regulation of m6A methylation modification is usually associated with the abnormal expression of regulators. Herein, we screened m6A regulators, and methylase RBM15 was found to be positively correlated with the severity of COVID-19, which corresponded with the proportion of genes with increased methylation levels, implying that RBM15 has an important role in SARS-CoV-2 infection by influencing methylation levels of genes. In addition, knockdown of RBM15 attenuated the elevation in both expression levels and methylation levels of CASP1, CASP5, IL17RB, TSLP, and TRIB1 stimulated by SARS-CoV-2 infection. These results indicated that RBM15 regulates the m6A levels of CASP1, CASP5 IL17RB, TSLP, and TRIB1 and consequently inducing an aberrant immune response and lymphopenia in COVID-19. The mechanism of target genes regulating cell death pathway and immune response pathway needs further explored.

In conclusion, this study identified RBM15 regulates the expression of multitarget genes by elevating m6A modification levels, aggravates the inflammatory response, and promotes cell death signaling pathways in COVID-19. The combined action of multiple targets has an important role in the pathogenesis of COVID-19, which explains why it is challenging to design a highly effective single-target therapy as well as the lack of specific drugs to combat COVID-19. Future studies are warranted to explore methods for inhibiting RBM15 expression, with the aim of identifying new treatment strategies for COVID-19.

## Materials and methods

### Patients

Blood samples were collected from 39 patients and 20 healthy controls, the demographic characteristics of these patients, and healthy controls are provided in Supplementary Table 3. We selected PBMC from six patients ranged from ages 51–60 years old, with a median of 58 years old in the severe group (S), a median of 49 years old in the mild group (M), and a median of 55 years old in the healthy controls (N) send to m6A microarray. The characteristics of these donors are provided in Supplementary Table 1. The study was approved by the medical ethics committee of the First Affiliated Hospital, College of Medicine, Zhejiang University; it conformed to the ethical guidelines of the Helsinki Declaration.

### Western blot

Cells were lysed using radioimmunoprecipitation assay buffer (50 mM Tris, pH 7.5, 120 mM NaCl, 1% Triton X-100, 0.5% sodium deoxycholate, 0.1% sodium dodecyl sulphate (SDS), 5 mM ethylenediaminetetraacetic acid) with 1% cocktail (Sigma, P2714) for 15 min. The supernatant was collected after centrifuge and then boiled with 5× loading buffer. Equal amounts of proteins were loaded and separated by SDS-polyacrylamide gel electrophoresis, transferred to nitrocellulose membranes (Millipore, HATF00010). The membranes were incubated with primary and secondary antibodies and subsequently incubated using WesternBright ECL kit for chemiluminescent reading on Bio-Rad. Antibodies used for western blot are as follow:

CASP1 (3866 T), cleaved–CASP3 (9664), and GAPDH (5174) were purchased from Cell Signaling Technology (CST) in MA, USA. CASP5 (DF7664), DDX3X (DF7429), TSLP (DF8077), and IL17RB (DF2510) were purchased from Affinity in Jiangsu, China. TRIB1 (K006294P) was purchased from Solarbio in Beijing, China.

### Cell lines

HuT 78 and THP-1 were obtained from the Cell Bank of Type Culture Collection of the Chinese Academy of Sciences. HuT 78 cells were cultured in Iscove’s Modified Dulbecco’s Medium (GIBCO, NY, USA), supplemented with 20% fetal calf serum (GIBCO, NY, USA). THP-1 cells were cultured in RPMI-1640 medium (GIBCO, NY, USA) supplemented with 10% fetal calf serum. All cells were cultured at 37 °C in a humidified atmosphere with 5% CO_2_.

### Construction of In vitro cell model

THP-1 was treated with 100 ng/ml phorbol 12-myristate 13-acetate, which was followed stimulated with or without 50 ng/ml Spike-ECD for 24 h. Cells were then co-cultured with HuT 78 cell line for another 24 h. For siRBM15 cell model, siNC and siRBM15 segments were transiently transfected with Lipofectamine® 2000 to HuT 78 cells separately and transfection reagents were removed after 8 h. Activated and stimulated THP-1 were then co-cultured with HuT 78 cells. Cells were harvest after 0, 6, 12, 24 h and underwent RNA extraction and qRT-PCR assay.

### CCK-8 assay

HuT 78 cells were cultured overnight on a 96-well plate (100 μL per well contains 2000 cells). The next day, the change medium contained 50 ng/ml spike-ECD for each well (100 μL per well). In all, 10 μL CCK-8 solution was added after 0, 12, 24 h. The culture medium with 1% CCK-8 solution was used as a blank control group. The absorbance at 450 nm was measured 4 h after added CCK-8.

### Analysis of iCLIP-Seq and MeRIP-seq data

iCLIP-seq data are with the following accession numbers: GSE63753 [28] and GSE78030 [18]. MeRIP-seq data were obtained from GEO data sets [29, 30]. All data were aligned to the corresponding reference genome and visualized using IGV: Integrative Genomics Viewer (http://www.broadinstitute.org/igv).

### Preparation of PBMC

PBMC were obtained from the whole blood of COVID patients and healthy donors at the First Affiliated Hospital, College of Medicine, Zhejiang University. Peripheral blood sample (at least 4 ml) was drawn into vacutainer tubes then separate PBMC by the Ficoll density gradient centrifugation method. We add an equal amount of phosphate-buffered saline (PBS) into the peripheral blood. Transferred it to the ficoll tube then centrifugated at 1000 × *g* for 15 min at room temperature. The buffy coat of PBMC cells was pooled and collected into a 15 ml centrifuge tube. Add 10 ml PBS to wash the PBMC cells and centrifuge at 250 × *g* for 10 min at room temperature twice. Collect the precipitate and transferred 1 ml Trizol reagent (Invitrogen, Carlsbad, CA, USA) into samples then frozen in liquid nitrogen immediately for later mRNA extraction.

### RNA extraction and quality control

Total RNA from each sample was extracted using Trizol reagent (Invitrogen, Carlsbad, CA, USA) and following the manufacturer’s instructions. The purity and amount of total RNA samples were determined with NanoDrop ND-1000 (Thermo Fisher, Shanghai, China).

### M6A immunoprecipitation

Total RNA (3 μg) each of nine samples (three severe cases, three mild cases, and three healthy controls) and m6A spike-in control mixture was transferred into an RNase-Free tube and labeled. Add 2 μg anti-m6A rabbit polyclonal antibody (Synaptic Systems, Goettingen, Germany) to 300 μl prepared 1× IP buffer (50 mM Tris-HCl, pH 7.4, 150 mM NaCl, 0.1% NP40, 40 U/μl RNase inhibitor) with sufficient mixing. The reaction was incubated with head-over-tail rotation at 4 °C for 2 h after added IP buffer with the m6A antibody. 20 μl Dynabeads™ M-280 Sheep Anti-Rabbit IgG suspension per sample was blocked with 0.5% bovine serum albumin at 4 °C for 2 h, then added 1× IP buffer washed the 30 s repeated three times and resuspended in the RNA-m6A-antibody mixture. After incubated with head-over-tail rotation at 4 °C for 2 h, the beads were then washed with 500 μl 1× IP buffer three times and twice with 500 μl wash buffer (50 mM Tris-HCl, pH 7.4, 50 mM NaCl, 0.1% NP40, 40 U/μl RNase inhibitor). Transferred supernatant as “sup”. Add 200 μl elution buffer (10 mM Tris-HCl, pH 7.4, 1 mM ethylenediaminetetraacetic acid, 0.05% SDS, 40 U proteinase K) into beads incubate at 50 °C for 1 h. After eluted, the supernatant was transferred as “IP”. RNA was extracted by acid phenol-chloroform and ethanol precipitated.

### M6A-mRNA and lncRNA epitranscriptomic microarray analysis

Arraystar Super RNA Labeling Kit was used to label the “IP” and “Sup” RNAs with Cy5 and Cy3, respectively. The labeled RNAs were hybridized onto Arraystar Human mRNA&lncRNA Epitranscriptomic Microarray (8x60K, Arraystar). Arrays were scanned in two-color channels by an Agilent Scanner G2505C (Agilent, Beijing, China) after washing the slides three times. Agilent Feature Extraction software (version 11.0.1.1) was used to analyze the acquired array images. Raw intensities of the IP (immunoprecipitated, Cy5-labeled) and Sup (supernatant, Cy3-labeled) were normalized with the average of the log2-scaled spike-in RNA intensities. After spike-in normalization, the probe signals having present (P) or marginal (M) QC flags in at least three out of nine samples were retained as “All Targets Value” in an Excel sheet for further “m6A methylation level” and “m6A quantity” and “expression level” analyses. “m6A methylation level” was calculated for the percentage of modification based on the IP (Cy5-labeled) and Sup (Cy3-labeled) normalized intensities. “m6A quantity” was calculated for the m6A methylation amount based on the IP (Cy5-labeled) normalized intensities. “expression level” was calculated based on the total of IP (Cy5-labeled) and Sup (Cy3-labeled) normalized intensities of RNA. Differentially m6A-methylated RNAs or differentially expressed RNAs between two comparison groups were identified by filtering with the FC and statistical significance (*p* value) thresholds. Hierarchical Clustering was performed to show the distinguishable m6A methylation or expression pattern among samples. Microarray analyses were performed by Kang Chen Biotech (Shanghai, China).

### GO and pathway analysis (GSEA analysis)

The GO analysis was performed for hypermethylated as wells as upregulated mRNA transcripts using the Metascape (http://metascape.org) platform. GO annotation analysis on the targets was performed and the results were saved and sorted by the number of targets involved in each entry to screen top biological processes and pathways. The significant *p* value of the pathway correlated is less than 0.01. GSEA is a computational method that determines whether an a priori defined set of genes shows statistically significant, concordant differences between two biological states. GSEA was performed using normalized data by GSEA_4.1.0 tool (http://software.broadinstitute.org/gsea/index.jsp). To explore the differences in potential functions in the hypermethylated with upregulated gene sets from severe cases and mild cases. GSEA was used using the Molecular Signatures Database (MSigDB) of Hallmarks gene sets (h.all.v7.1.symbols), Canonical pathways gene sets (c2.cp.v7.1.symbols) and GO biological process gene sets (c5.bp.v7.1.symbols).

### Quantitative real-time PCR

After extraction, total RNA was reverse-transcribed to cDNA using PrimeScript RT Reagent Kit (Takara, Dalian, China) according to the manufacturer’s instructions. RT-qPCR was performed using SYBR Select Master Mix (Invitrogen, Carlsbad, CA, USA) as recommended by the manufacturer. GAPDH was used as the normalization control and the relative expression levels were calculated using the 2^−ΔΔCt^ method. The primers were listed in Supplementary Table 4.

### siRNA transfection

The siRNA specifically targeting RBM15 was synthesized by Sangon Biotech (Shanghai, China) “RBM15-1: GGAAGAAAGCTAATCTGTTTAGTAT, huRBM15-2: GCAGCGG AAAGACCGATAGCGGCGG”. Target cells were transiently transfected with Lipofectamine® 2000 transfection reagent (Invitrogen, CA, USA) according to the instructions.

### m6A quantification

The change of global m6A levels in total mRNA was measured by EpiQuik m^6^A RNA Methylation Quantification Kit (Colorimetric) (Epigentek) following the manufacturer’s instructions. In all, 200 ng RNA was used to analysis, three parallel controls for each sample.

### Apoptosis status

The cell death ratio was analyzed using the Annexin V-FITC Apoptosis Detection Kit (Beyotime, Shanghai, China). At 24 h after stimulated with Spike protein, cells were harvested and resuspended in binding buffer containing Annexin V-FITC (0.01 mg/mL) and PI (1 mg/mL) according to the manufacturer’s instructions. The samples were analyzed by flow cytometry (BD Biosciences, USA). Cells were discriminated into viable cells, necrotic cells, and apoptotic cells by using BD FACSDiva 6.1.3 software (BD Biosciences, USA), and then the percentages of necrotic and apoptotic cells from each group were compared. Experiments were performed three times.

### Statistical analysis

For m6A microarray analysis and qPCR, the significance of differences in methylation level and methylation quantity between the healthy control and patient groups was evaluated with an unpaired two-sided *t* test. For GO and KEGG analyses, Fisher’s exact test was applied to evaluate the significance of GO terms and KEGG pathway identifier enrichment in gene sets with hypermethylation as well as upregulated mRNAs.

## Supplementary information

Supplementary figure legends

Supplementary Figure 1

Supplementary Figure 2

Supplementary Table 1

Supplementary Table 2

Supplementary Table 3

Supplementary Table 4
