# Supplementary material for: RBM15-mediated N6-methyladenosine modification affects COVID-19 severity by regulating the expression of multitarget genes
Source: Cell Death Dis. 2021 Jul 23;12(8):732. doi: 10.1038/s41419-021-04012-z (PMC8298984; doi:10.1038/s41419-021-04012-z)
Supplement: Supplementary file 1 — Supplementary figure legends [file 41419_2021_4012_MOESM1_ESM.docx]

**Supplementary figure legends**

**Supplementary Figure 1:** (A) and (B) showed the principal component analysis of m6A methylation level and mRNA expression level. (C) Volcano plot showing abnormally methylated mRNAs in group S (left) and M (right), as compared with group N (FC > 1.5, P < 0.05). And Venn diagram showed the number of hyper- and hypo- methylated genes in S vs. N and M vs. N. (D) Scatter plot of differentially expressed mRNAs in S and M separately compared with N. mRNAs above the top line and below the bottom line showed a significant change in expression levels (FC > 1.5, P < 0.05)

**Supplementary Figure 2:** (A) The top GO annotation results of up to 20 enriched clusters. (B) A map of the top 20 groups of enrichment results. (C) Volcano plots of log2 FC of upregulated transcripts (FC > 1.5, P < 0.05) and hyper-methylated transcripts (FC > 2, P < 0.05) in group S in comparison with group N.(D) The expression levels and m6A enrichment levels of SERPINA7 in patients. (E) CASP1, CASP5, DDX3X, TRIB1, IL17RB, and TLSP expression levels in HuT 78 cells cocultured with activated THP-1 cells were measured after treated with HA proteins separately for 0, 6, 12, and 24 h.
