# Supplementary figures and images for: RBM15-mediated N6-methyladenosine modification affects COVID-19 severity by regulating the expression of multitarget genes
Source: Cell Death Dis. 2021 Jul 23;12(8):732. doi: 10.1038/s41419-021-04012-z (PMC8298984; doi:10.1038/s41419-021-04012-z)

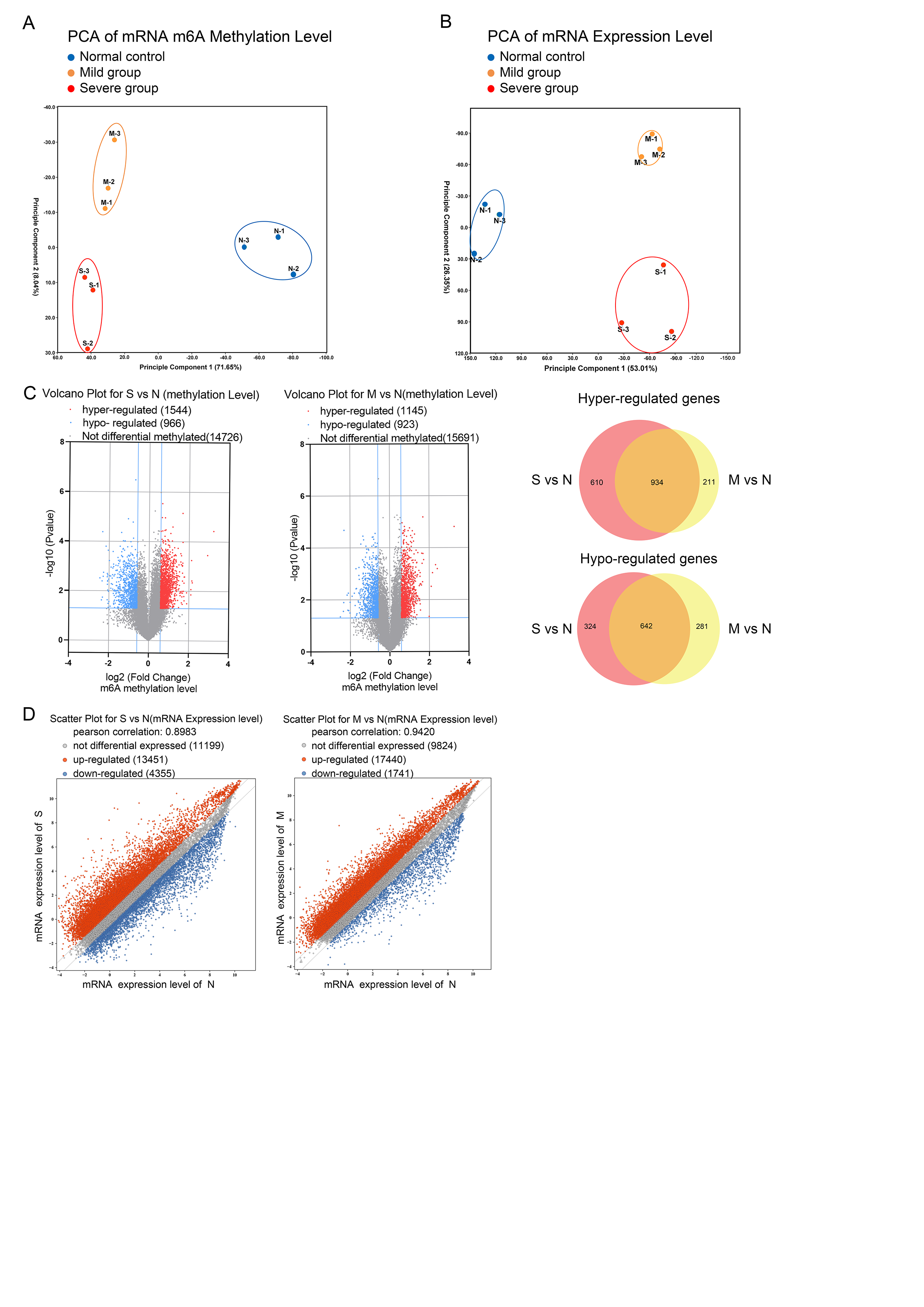

Supplement: Supplementary file 2 — Supplementary Figure 1 [file 41419_2021_4012_MOESM2_ESM.tif]

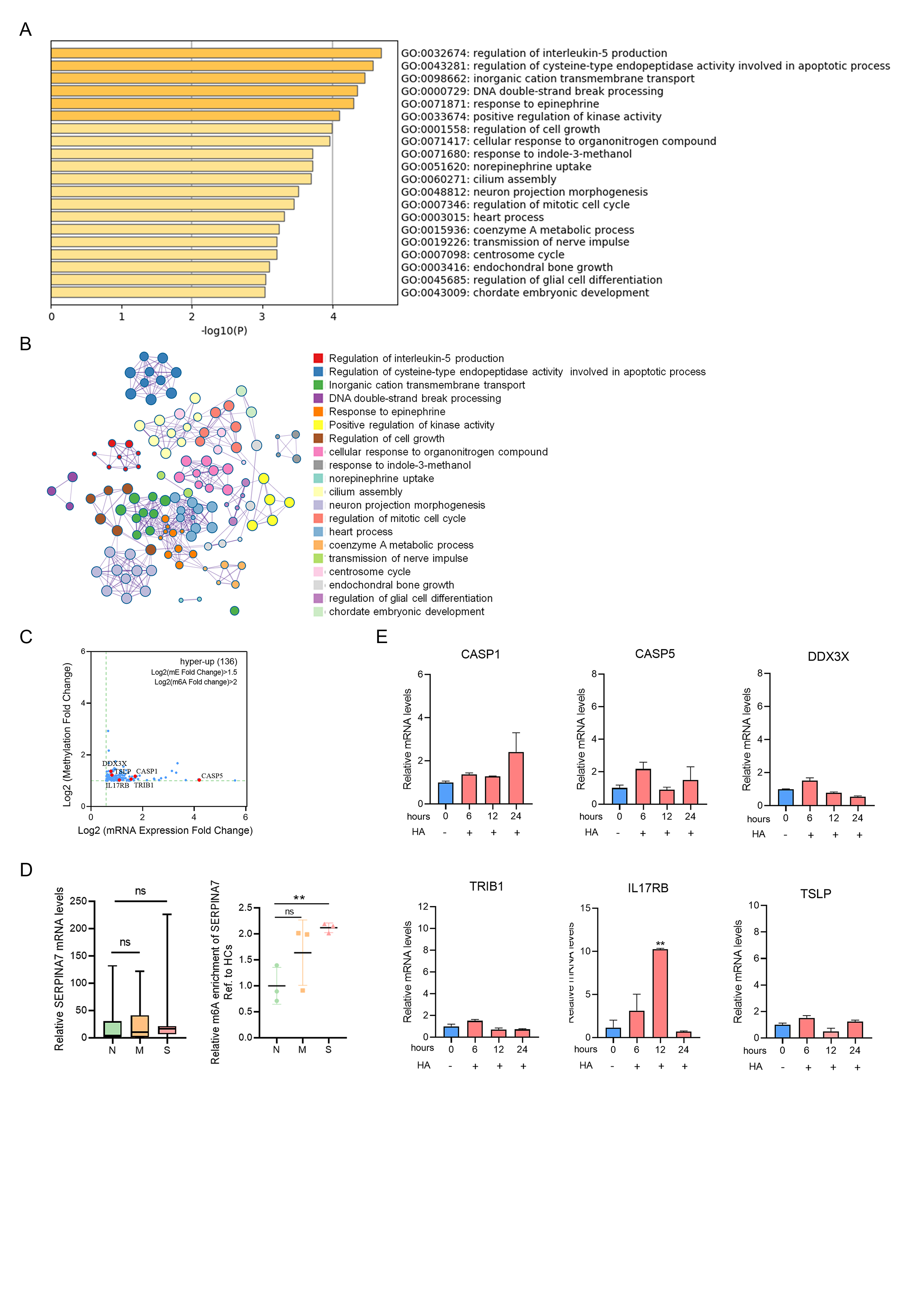

Supplement: Supplementary file 3 — Supplementary Figure 2 [file 41419_2021_4012_MOESM3_ESM.tif]
